# Ryanodine Receptors in Islet Cell Function: Calcium Signaling, Hormone Secretion, and Diabetes

**DOI:** 10.3390/cells14100690

**Published:** 2025-05-10

**Authors:** Md. Shahidul Islam

**Affiliations:** 1 Karolinska Institutet, Department of Clinical Sciences and Education, Södersjukhuset, Research Center, 5th Floor, SE-118 83 Stockholm, Sweden; shahidul.islam@ki.se; Tel.: +46-70-259-3446; 2 Department of Internal Medicine, Uppsala University Hospital, SE-751 85 Uppsala, Sweden

**Keywords:** ryanodine receptors in islets cells, calcium-induced calcium release in islet cells, ryanodine receptors and insulin secretion, calcium signaling in beta cells, endoplasmic reticulum stress in beta cells, glucagon-like peptide-1 and ryanodine receptors, ryanodine receptors in delta cells, beta-cell electrical activity and ryanodine receptors, ryanodine receptors and diabetes, ryanodine receptors and store-operated calcium entry

## Abstract

Ryanodine receptors (RyRs) are large intracellular Ca^2+^ release channels primarily found in muscle and nerve cells and also present at low levels in pancreatic islet endocrine cells. This review examines the role of RyRs in islet cell function, focusing on calcium signaling and hormone secretion, while addressing the ongoing debate regarding their significance due to their limited expression. We explore conflicting experimental results and their potential causes, synthesizing current knowledge on RyR isoforms in islet cells, particularly in beta and delta cells. The review discusses how RyR-mediated calcium-induced calcium release enhances, rather than drives, glucose-stimulated insulin secretion. We examine the phosphorylation-dependent regulation of beta-cell RyRs, the concept of “leaky ryanodine receptors”, and the roles of RyRs in endoplasmic reticulum stress, apoptosis, store-operated calcium entry, and beta-cell electrical activity. The relationship between RyR dysfunction and the development of impaired insulin secretion in diabetes is assessed, noting their limited role in human diabetes pathogenesis given the disease’s polygenic nature. We highlight the established role of RyR-mediated CICR in the mechanism of action of common type 2 diabetes treatments, such as glucagon-like peptide-1, which enhances insulin secretion. By integrating findings from electrophysiological, molecular, and clinical studies, this review provides a balanced perspective on RyRs in islet cell physiology and pathology, emphasizing their significance in both normal insulin secretion and current diabetes therapies.

## 1. Introduction

Ca^2+^ signaling is important for the secretion of various hormones from the endocrine cells in the islets of Langerhans [1,2]. In these cells, Ca^2+^ signaling is generated mainly by two mechanisms: (1) Ca^2+^ entry through the Ca^2+^ channels located on the plasma membrane and (2) Ca^2+^ release from the endoplasmic reticulum (ER) via the intracellular Ca^2+^ release channels, specifically the inositol 1,4,5-trisphosphate receptors (IP3Rs) and the ryanodine receptors (RyRs). In islet endocrine cells, IP3Rs are more abundant than RyRs [3,4]. Ca^2+^-induced Ca^2+^ release (CICR) is a process whereby an increase in the cytosolic free Ca^2+^ concentration ([Ca^2+^]_i_) triggers more Ca^2+^ releases from the ER [5]. IP3Rs can mediate CICR in the presence of inositol 1,4,5-trisphosphate (IP3) [6,7,8]. Compared to IP3Rs, RyRs are more effective for CICR because Ca^2+^ can directly activate RyRs, and RyRs have a much higher Ca^2+^ conductance than IP3Rs [9,10]. The conductances of RyRs and IP3Rs with K^+^ as the charge carrier are ~700 and ~80 pS, respectively [10,11]. However, RyRs are much less abundant in islet cells, and partly for this reason, some investigators have found it difficult to demonstrate RyR-mediated CICR in these cells.

Since the discovery of CICR in 1992, more than three decades of research have generated enormous information about the roles of this process and RyRs in the regulation of Ca^2+^ signaling and secretion, especially in β-cells [12]. A head-to-head comparison between the relative importance of IP3Rs and RyRs in islet cell physiology is not the aim of this review. Instead, I will summarize the knowledge generated in recent years about the roles of CICR and RyRs in islet cells. I will also explain the potential reasons for the discrepancies in the results obtained by various researchers.

## 2. Effects of Different Agonists and Antagonists on RyRs

Investigators have used many exogenous or endogenous agonists and antagonists of RyRs to study CICR and RyRs in β-cells [13]. Interpretation of the results obtained with some of these pharmacological tools can sometimes be difficult, since many of these tools have off-target effects. Appropriate experimental protocols and cautious interpretations are necessary to avoid misleading conclusions. In the following paragraphs, I will discuss some of the agents used for studying RyRs and CICR in islet cells.

### 2.1. Caffeine

Caffeine is the most commonly used pharmacological tool for activating RyRs. In insulin-secreting cells, caffeine triggers Ca^2+^ release from the ER under some experimental conditions [6,14,15,16,17,18,19,20], whereas it fails to do so in other conditions [12,21,22,23,24,25]. In intact β-cells, caffeine releases Ca^2+^ from the ER in some experiments [25,26,27], whereas it fails to do in others [21,22,23]. Many factors may account for these differences, including the filling state of the ER Ca^2+^ store, the phosphorylation status of the RyRs [28], and the experimental conditions used by different investigators.

There are some difficulties in interpreting the caffeine-induced increase in [Ca^2+^]_i_ in intact β-cells. For instance, caffeine inhibits the K_ATP_ channel, leading to membrane depolarization and subsequent Ca^2+^ entry through voltage-gated Ca^2+^ channels (VGCCs) [22]. Caffeine also inhibits cAMP-phosphodiesterase enzymes, making it difficult to determine whether the effects of caffeine are mediated by caffeine itself or by increased cAMP [29].

### 2.2. Ryanodine

Ryanodine has been used for studying RyRs for over four decades, and so far, no notable off-target effects have been reported. Low concentrations of ryanodine open the channel, and high concentrations close the channel, but the channel can stay in different conformations other than in a simple “open” or “closed” state [30]. Ryanodine alters the open probability, conductance, and gating of the channel by binding to the high- and low-affinity sites of the channel [31]. At nanomolar concentrations, it binds to a single high-affinity site located within the pore of the channel, thereby increasing the open probability but reducing the conductance of the channels [32]. At micromolar concentrations, it binds to both the high-affinity site and one or more low-affinity sites, thereby locking the channel in a closed state [33]. In experiments, the actual concentrations at which ryanodine activates or inhibits RyRs may depend on numerous factors, such as whether it is being used in a cell-free system, permeabilized cells, intact cells, lipid bilayers, single cells, clusters of cells, tissue slices, and many other experimental conditions.

Since ryanodine can both activate and inhibit the channel, it may sometimes be difficult to interpret the results obtained from experiments in which ryanodine is used. For instance, while a high concentration of ryanodine inhibits RyRs during washout, one may see the stimulatory effect of ryanodine [34]. Investigators may need to try out different concentrations of ryanodine and different incubation times or follow the experimental protocols that have worked for other investigators in similar experiments. The use of inappropriate experimental protocols in experiments where ryanodine was used to inhibit RyRs has led to some misleading conclusions [35,36,37,38].

#### 2.2.1. Activation of RyRs by Ryanodine

An increase in [Ca^2+^]_i_ in any cell due to nanomolar concentrations of ryanodine is a highly reliable sign of the existence of functional RyRs in the cell. In dispersed human β-cells, nanomolar concentrations of ryanodine increase [Ca^2+^]_i_ and insulin secretion [36]. In fresh mouse pancreatic tissue slices, activation of RyRs in β-cells by 100 nM ryanodine induced [Ca^2+^]_i_ increase of short durations in a regenerative fashion [34].

#### 2.2.2. Inhibition of RyRs by Ryanodine

To inhibit RyRs in β-cells, investigators have successfully used ryanodine at concentrations ranging from 10 to 400 μM and have used different experimental protocols [6,20,35,39,40,41]. When used at very high concentrations (e.g., >100 μM), ryanodine can inhibit channels completely in a relatively short time [13,34]. Ryanodine inhibits RyRs in a use-dependent manner [42]. Use-dependent means that ryanodine binds preferentially to open-state channels. To inhibit RyRs, it may be necessary to treat cells with high concentrations of ryanodine, often for prolonged periods. The kinetics of association of ryanodine with RyRs is slow, with the half-time for the association rate being as slow as 36 min [43]. For this reason, it may be necessary to incubate cells in 200 μM ryanodine for two hours [18]. The use of lower concentrations of ryanodine for shorter periods has led to the conclusion that ryanodine cannot inhibit glucose-induced increase in [Ca^2+^]_i_ and insulin secretion [36]. In experiments where whole islets are used, it may take several hours to achieve sufficiently high concentrations of ryanodine inside the islets. Using the fluorescent ryanodine analog BODIPY FL-X ryanodine, Llanos et al. showed that it may take as long as 12 h for ryanodine concentration to increase to a sufficiently high level in all islet cells [35]. To inhibit insulin secretion from islets, it is necessary to incubate the islets in 200 μM ryanodine for as long as 12 h [35]. Ryanodine does not inhibit VGCCs and does not damage the cells [20,35]. Careful attention to experimental protocols is necessary to demonstrate the inhibitory action of ryanodine on RyR-dependent processes [20,39,44,45,46].

When used at high concentrations, the binding of ryanodine to RyRs is usually irreversible in the time frame of single-channel experiments. However, in the time frame of the experiments reported by Postić et al., the activation was reversible on washout of ryanodine [34]. Apparently, in intact cells, ryanodine can be inactivated presumably by cytochrome P450 enzymes.

### 2.3. 9-Methyl-7-bromoeudistomin D (MBED)

MBED is about 1000 times more potent than caffeine and activates RyRs in a caffeine-like manner [47]. MBED increases [Ca^2+^]_i_ and stimulates insulin secretion from insulin-secreting cells [42,48]. Unlike caffeine, MBED does not inhibit cAMP-PDE activity in these cells [48]. However, MBED is not readily available from commercial sources.

### 2.4. Thimerosal

Thimerosal, a sulfhydryl oxidizing agent, activates RyRs by interacting with critical thiol groups associated with the channel [49]. It releases Ca^2+^ from the ER by activating RyRs in RINm5F cells, MIN6 cells, and mouse islet cells [12,25,50]. Thimerosal can also activate IP3Rs, but these cells express inositol 1,4,5-trisphosphate receptor, type 3 (IP3R3) [51], which is not activated but rather inhibited by thimerosal [52].

### 2.5. Dantrolene

Dantrolene, even at low concentrations (e.g., 10 μM), inhibits RyR1 and RyR3 [53]. In β-cells that express RyR1, glucose-induced [Ca^2+^]_i_ oscillations are inhibited by 10 μM dantrolene [54]. In MIN6 cells, which express both RyR1 and RyR2, harmane-induced Ca^2+^ increase and insulin secretion are inhibited by 10 μM dantrolene [25]. Glucose-induced insulin secretion from rat or mouse islets is inhibited by 10 μM dantrolene [55,56], but 10 μM dantrolene does not inhibit insulin secretion from human islets [36].

To inhibit RyR2, high concentrations of dantrolene are usually needed, but if RyR2 becomes phosphorylated, the channel becomes more susceptible to inhibition by dantrolene [57,58,59]. Since human β-cells mainly express RyR2 [3], it is not surprising that glucose-induced insulin secretion from human β-cells is not inhibited by 10 μM dantrolene [36]. In insulin-secreting INS-1E cells, which also mainly express RyR2, insulin secretion mediated by RyR2-mediated CICR is inhibited by a high concentration (e.g., 75 μM) of dantrolene [48]. Dantrolene is only slightly soluble in water. For experiments requiring high concentrations, such as 100 μM, it is recommended to freshly dissolve dantrolene in polyethylene glycol 600 before each experiment [55].

It is possible that alternative splicing of RyR2 may determine the channel’s sensitivity to inhibition by dantrolene. β-cells express the “islet type” RyR2 that is generated by alternative splicing of exons 4 and 75 [60]. The sensitivity of this splice variant to inhibition by dantrolene is unknown.

Dantrolene may have broader effects, and some of its effects are poorly understood. For instance, it can bind to the IP3-binding domain of IP3Rs [61] and inhibit IP3-induced Ca^2+^ increase in some cells [62]. A paradoxical effect of dantrolene, characterized by an increase in insulin secretion through mechanisms that are not yet fully understood, has also been described [63,64]. Another effect of high concentrations of dantrolene is the inhibition of glucose oxidation, which can complicate the interpretation of the insulin secretion data [55].

### 2.6. Other Agonists of RyRs

#### 2.6.1. 4-Chloro-m-cresol (4-CmC) and 4-Chloro-3-ethylphenol (4-CEP)

4-Chloro-m-cresol (4-CmC) is a potent and clinically relevant activator of RyR1, but when used at high concentrations, it can also activate RyR2 and RyR3 [65,66,67]. 4-Chloro-3-ethylphenol (4-CEP), which has a more hydrophobic ethyl group instead of a methyl group at the 3-position, is more bioactive [66]. These agents do have some off-target effects; for instance, they inhibit the ORAI1-3 channels [68]. We and other investigators have reported that 4-CmC and 4-CEP activate RyRs in insulin-secreting cells [20,28,69,70,71].

#### 2.6.2. Nitric Oxide (NO)

NO can activate RyRs either directly or through oxidation or poly-S-nitrosylation of critical thiol groups associated with the channel [13,72]. Glucose stimulates the formation of NO in β-cells [73,74]. Low concentrations of gaseous NO increase [Ca^2+^]_i_ by activating RyRs and stimulate insulin secretion from rat β-cells [18].

#### 2.6.3. Arachidonic Acid

Previous studies have shown that arachidonic acid releases Ca^2+^ from the sarcoplasmic reticulum through the activation of RyRs [75,76]. Arachidonic acid increases [Ca^2+^]_i_ in β-cells by activating the RyRs [39]. In this context, it is noteworthy that glucose stimulation increases arachidonic acid in β-cells [77].

## 3. Role of RyRs in Mediating CICR in β-Cells

By using a variety of methods, many groups have confirmed that β-cells express RyRs, but their expression levels are lower than in many other tissues [14,28,44,54,78]. The presence of RyRs in β-cells has been confirmed by RNAse protection assay, real-time quantitative polymerase chain reaction, and RNA sequencing [3,28,44,54,78]. RyRs are more effective than IP3Rs in mediating CICR because they can be directly activated by Ca^2+^, and in the physiological range of [Ca^2+^]_i_, RyRs act solely as Ca^2+^-activated channels [10,11]. RyRs also have very high conductance and thus are more effective than IP3Rs in amplifying Ca^2+^ signals through CICR [11].

An increase in [Ca^2+^]_i_ caused by Ca^2+^ entry through VGCCs triggers CICR through RyRs in β-cells [14,20,44,46]. However, this activation is not like the activation of RyRs and CICR in skeletal muscle or heart, where VGCCs and RyRs are distributed in an orderly dyadic or triadic pattern [79]. Thus, in single mouse β-cells, a 100 ms depolarizing voltage clamping step does not always trigger CICR [80]. Unlike in the heart, where RyRs are closely coupled to and rapidly activated by VGCCs during each action potential, β-cells appear to have a “loose” coupling, similar to smooth muscle cells [81]. This is likely due to the low abundance of RyRs in β-cells. Consequently, RyR activation in β-cells seems to depend on a global increase in [Ca^2+^]_i_ resulting from VGCC-mediated Ca^2+^ influx rather than a localized, tightly coupled interaction between VGCCs and RyRs.

There is no certainty that an increase in [Ca^2+^]_i_ caused by depolarization-induced Ca^2+^ entry through VGCCs will always trigger substantial CICR in β-cells. Usually, a sufficiently high [Ca^2+^]_i_ is required for activating RyRs [10]. The density of VGCCs in β-cells is low [34,82]. Depolarization of β-cells upon stimulation by glucose alone often leads to an increase of [Ca^2+^]_i_ to only about 300 nM. This concentration of [Ca^2+^]_i_ is usually not sufficient for triggering CICR. However, such concentrations of cytoplasmic Ca^2+^ can initiate CICR in the presence of cAMP generating agents, high luminal Ca^2+^ concentration, and positive modulatory factors of RyRs like ATP, NO, glycolytic intermediates, and arachidonic acid [13].

## 4. Magnitude of [Ca^2+^]_i_ Increase Achieved Through RyR-Mediated CICR

Numerous studies have measured [Ca^2+^]_i_ in β-cells upon glucose stimulation using fluorescent Ca^2+^ indicators. Some studies report raw fluorescence changes implying [Ca^2+^]_i_ changes, while others calibrate fluorescence signals to [Ca^2+^]_i_ changes using different methods. These studies show that at low glucose concentrations, [Ca^2+^]_i_ is about 100 nM. Upon high glucose stimulation, [Ca^2+^]_i_ typically increases three-fold. This relatively modest increase may be due to experimental conditions not supporting the optimal engagement of the CICR process.

CICR amplifies Ca^2+^ signals in β-cells to varying degrees depending on the experimental conditions [28,42,46]. The magnitude of the global [Ca^2+^]_i_ increase at the peak of CICR depends on how effectively the process is engaged. Agents that increase cAMP play a crucial role in this process [28]. In the presence of cAMP-elevating agents, peak [Ca^2+^]_i_ increase during the CICR upstroke can reach high levels, typically 0.5–2 μM [6,42,83].

Optimal stimulation of β-cells by nutrients can likely increase [Ca^2+^]_i_ to very high levels, potentially around 10 μM. Such high [Ca^2+^]_i_ levels are achieved through CICR and the fusion of multiple Ca^2+^ events. However, these high concentrations cannot be detected by commonly used high-affinity Ca^2+^ indicators. Low-affinity Ca^2+^ indicators like Calbryte are more suitable for measuring such high concentrations [84].

[Ca^2+^]_i_ increase caused by CICR often occurs in the form of large regenerative Ca^2+^ spikes of short duration [6,28,34,46]. Local [Ca^2+^]_i_ increases caused by CICR may be even higher, but measuring these local increases is challenging. Relatively high [Ca^2+^]_i_ increases are necessary for mediating certain cellular processes, such as 1. insulin exocytosis (requiring approximately 1 μM Ca^2+^) [85], and 2. activation of the large-conductance BK (KCa1.1) channels (requiring Ca^2+^ concentrations of around 10 μM [86].

These high [Ca^2+^]_i_ levels highlight the importance of CICR in β-cell function and the need for appropriate experimental techniques to accurately measure and study this process.

## 5. Regulation of RyRs in β-Cells by Phosphorylation

The regulation of RyRs in β-cells through phosphorylation is a critical process that influences Ca^2+^ signaling and insulin secretion. This section will focus on the effects of phosphorylation by two key kinases: Ca^2+^-calmodulin-dependent protein kinase II (CaMKII) and protein kinase A (PKA).

### 5.1. CaMKII-Mediated Phosphorylation

Glucose stimulation of β-cells activates CaMKII, leading to the phosphorylation of RyR2 [87]. This phosphorylation enhances the sensitivity of RyR2 to trigger Ca^2+^, thereby amplifying CICR [88]. The primary site for CaMKII-mediated phosphorylation on mouse RyR2 is the serine residue at position 2814 (S2814). Phosphorylation of S2814 increases RyR2 channel activity. When S2814 of mouse RyR2 is replaced by aspartic acid (D) (S2814D), it mimics constitutive pseudo-phosphorylation of RyR2 by CAMKII. In S2814D knock-in mice, the channel has a gain-of-function defect. In these mice, there is enhanced Ca^2+^ leak from the ER, reduced ER Ca^2+^ pool size, and decreased frequency and amplitude of glucose-stimulated Ca^2+^ oscillations in β-cells. These mice exhibit basal hyperinsulinemia, glucose intolerance, and reduced glucose-induced insulin secretion, closely resembling β-cells defects observed in human type 2 diabetes mellitus (T2DM).

Indeed, increased CaMKII-mediated phosphorylation at S2814 has been reported in the β-cells of individuals with T2DM [87]. It is conceivable that overstimulation of β-cells (e.g., due to overeating) leads to increased [Ca^2+^]_i_, chronic activation of CaMKII, and CaMKII-mediated hyperphosphorylation of RyR2. Chronic activation of RyR2 by CaMKII leads to Ca^2+^ leak, resulting in higher basal insulin secretion and impairment of glucose-induced insulin secretion.

### 5.2. Phosphorylation by PKA

The primary site for the PKA-mediated phosphorylation of RyR2 is serine at 2808 (S2808) [64,89]. In β-cells, PKA-mediated phosphorylation of RyR2 is essential for the functional recruitment and activation of the channel by its agonists [28,90]. This phosphorylation appears to play a crucial role in overcoming the inhibitory effects of cytoplasmic Mg^2+^ on RyR2 [91]. PKA phosphorylation also enhances the sensitivity of RyR2 to the stimulatory effects of cytoplasmic Ca^2+^, thereby promoting CICR from the ER [44,64,92]. While the consequences of CaMKII-mediated hyperphosphorylation on RyR2 in β-cells are well established, the effects of PKA-mediated hyperphosphorylation require further investigation.

## 6. FK506-Binding Protein 12.6 (FKBP12.6) and RyR2

FKBP12.6 (product of *FKBP1B* gene), also known as calstabin2, binds to and stabilizes the closed state of RyR2, reducing the probability of channel opening. Its dissociation from RyR2 increases the excitability of the channel. One study has demonstrated that cADPR binds to FKBP12.6, thereby releasing Ca^2+^ through RyR2 in rat islet microsomes [93]. While several subsequent studies have investigated the functional relationship between cADPR, and FKBP12.6, no other study has directly replicated the binding assay. The view that cADPR is the endogenous ligand for FKBP12.6 remains controversial and not widely accepted.

Chen et al. have shown that in Fkbp1b knock-out mice, glucose-induced [Ca^2+^]_i_ increase in β-cells is enhanced, and consistent with this, glucose-induced insulin secretion is also increased [94]. FKBP12.6 has a stabilizing effect on RyR2, and in the absence of FKBP12.6, the channel becomes more sensitized to trigger by Ca^2+^, leading to enhanced CICR. On the other hand, Noguchi et al. reported dramatically different results. They showed that in their Fkbp1b knock-out mice, glucose-induced insulin secretion was markedly decreased [95].

The reasons for such contradictory results may be because in the study by Chen et al., exon 3 was deleted, and in the study by Noguchi et al., exon 1 was deleted. The different exon deletions effectively created distinct “splice variants” of Fkbp1b. Exon 1 deletion might lead to a more complete loss of FKBP12.6 function, leading to Ca^2+^ leak through RyR2, which can explain impaired insulin secretion [96]. Exon 3 deletion might result in a partially functional protein that increases RyR2 excitability in a way that enhances insulin secretion.

The contradictory findings could also be due to the different genetic backgrounds of the mouse models used (129/Sv/Ev mice in the study by Chen et al. and ICR mice in the study by Noguchi et al. It is possible that different genetic backgrounds trigger different levels of compensatory mechanisms and modify the expression of different genes involved in glucose metabolism and insulin secretion.

The dramatic alterations in insulin secretion observed in two different Fkbp1b knock-out mouse models, showing either increased or decreased secretion, suggest a significant role for RyR2-mediated CICR in regulating insulin secretion.

## 7. Cyclic ADP-Ribose (cADPR) and RyRs of β-Cells

cADPR plays a complex role in modulating RyRs, particularly RyR2. While cADPR can activate RyR2 directly [97], its primary function is to enhance RyR2’s sensitivity to Ca^2+^, thereby amplifying CICR [98]. Notably, cADPR does not bind directly to RyRs but interacts with them through intermediary proteins such as FKBP12.6 [93] and GAPDH [99].

In β-cells, whether cADPR releases Ca^2+^ from the ER depends on the specific cells, rodent models, experimental conditions, or labs performing the experiments. Some studies published in this field have not been replicated. cADPR does not activate RyRs in β-cells obtained from *ob/ob* mice or rat insulinoma cells [24,50,100].

Nevertheless, it is evident that glucose, especially in the presence of glucagon-like peptide-1 (GLP-1), increases cADPR level in β-cells [101]. Like several other small molecules, cADPR appears to sensitize RyRs in β-cells to enhance CICR [101]. Direct evidence that cADPR enhances CICR by sensitizing RyRs in β-cells is lacking, whereas evidence that cAMP does the same is robust [6,28,46,48].

## 8. Role of RyRs in Mediating Insulin Secretion

Ca^2+^ entry through VGCCs can cause a modest increase in [Ca^2+^]_i_, which is sufficient to trigger some insulin secretion. However, under certain conditions, this Ca^2+^ entry leads to CICR, resulting in a much higher [Ca^2+^]_i_. This elevated Ca^2+^ level substantially contributes to Ca^2+^-dependent insulin secretion.

The involvement of RyRs in glucose-stimulated insulin secretion is well established through both pharmacological and molecular experiments. Pharmacological agonists of RyRs, such as caffeine and MBED, enhance insulin secretion [47,48,102], while inhibitors like high concentrations of ryanodine and dantrolene suppress it [35,48,102]. Molecular studies have shown that deletion of RyR2 inhibits glucose-stimulated insulin secretion [103], and islets from knock-in mice with a RyR2 mutation exhibit impaired secretion [87]. Furthermore, humans and mice with a mutant leaky RyR2 also demonstrate impaired glucose-stimulated insulin secretion [96].

CICR mediates insulin secretion in a context-dependent manner, i.e., it increases insulin secretion only when the glucose concentration is high, and the RyRs are sensitized by exogenous agents like caffeine or endogenous modulators like cAMP [6,28,48].

The RyR2 isoform in β-cells differs from that in the heart [60]. Specifically, RyR2 mRNA in beta cells lacks both exon 4 and exon 75 [60]. This splice variant is more effective in mediating glucose-stimulated insulin secretion. Evidence for this comes from studies showing that insulin secretion is impaired in mice expressing the “exon 75-containing RyR2”, whereas it remains normal in mice expressing the “exon 75-deficient RyR2” [104].

## 9. Role of RyR-Mediated CICR in GLP-1-Induced Insulin Secretion

**Figure 1:**
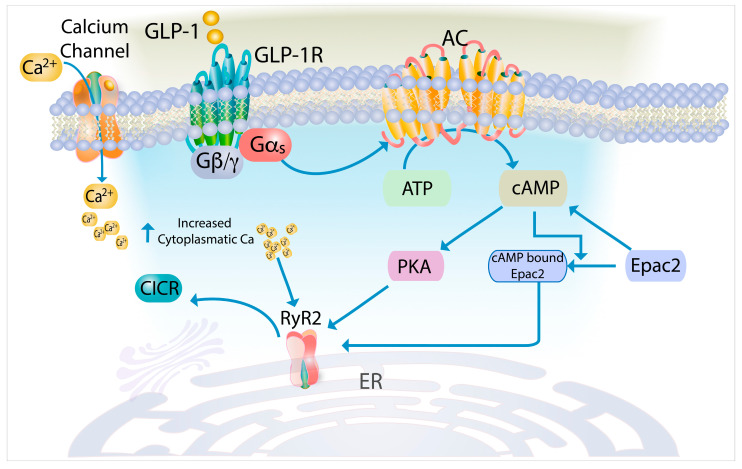
Schematic of GLP-1-mediated CICR in β-cells. The figure illustrates the sequential signaling events through which GLP-1 potentiates CICR. Binding of GLP-1 to its G-protein-coupled receptor activates Gα_s_ subunit, which stimulates adenylyl cyclase to generate cAMP. Elevated cAMP levels activate protein kinase A and bind to the exchange protein directly activated by cAMP 2 (Epac2). The cAMP-bound Epac2 directly interacts with RyR2, while PKA phosphorylates RyR2. These dual actions enhance RyR2 sensitivity to cytoplasmic Ca^2+^, resulting in enhanced CICR. Abbreviations: AC, adenylyl cyclase; Gα_s,_ stimulatory G-protein α-subunit; PKA, protein kinase A.

GLP-1 increases insulin secretion primarily at high glucose concentrations and halts secretion when glucose levels drop, thus preventing hypoglycemia [105]. Drugs that stimulate insulin secretion via the GLP-1 receptor and coupled G-protein activation are commonly used to treat T2DM. GLP-1 increases insulin secretion by generating cAMP, which affects various ion channels, including RyRs [106]. It has been established that GLP-1 stimulates insulin secretion by enhancing RyR-mediated CICR (Figure 1) [6,15,41,105,107].

GLP-1 sensitizes RyR2 through the protein kinase A (PKA) and the exchange protein activated by cAMP 2 (Epac2) pathways [90,108]. PKA phosphorylates RyR2 at Ser2809, increasing its open probability and sensitivity to cytoplasmic Ca^2+^ [28,109]. This phosphorylation also causes the dissociation of FKBP12.6, which stabilizes RyR2 in a closed state, thereby facilitating CICR [109].

GLP-1-induced cAMP directly activates Epac2, sensitizing RyR2 and promoting CICR [16,90]. However, the molecular mechanisms by which Epac2 enhances CICR via RyR2 remain unclear.

Through PKA activation, GLP-1 facilitates Ca^2+^ influx via VGCCs, increasing [Ca^2+^]_i_. This activates CaMKII, leading to RyR2 phosphorylation, which enhances their sensitivity to Ca^2+^ and further facilitates CICR [87].

GLP-1 also increases the formation of cADPR, and the GLP-1-induced Ca^2+^ increase is inhibited by ryanodine and 8-bromo-cADPR, an inhibitor of cADPR-induced Ca^2+^ signaling [101].

## 10. Link Between Glucose Metabolism and Activation of RyRs

The stimulation of β-cells by high glucose leads to diverse changes that are known to modulate the RyRs positively, enhancing CICR. For instance, glucose stimulation leads to an initial uptake of Ca^2+^ into the ER mediated by SERCA, increasing the filling state of the ER Ca^2+^ store [110]. Increased ER Ca^2+^ load increases the likelihood of activation of RyRs [111].

Glucose stimulation increases the concentration of cytoplasmic ATP, which is known to enhance Ca^2+^-induced activation of RyR2 [35,112]. Increased ATP binds to free Mg^2+^ ions, effectively reducing the concentration of free Mg^2+^ in the cytosol [113]. Mg^2+^ is an inhibitor of RyRs [114]. Therefore, the reduction in free cytosolic Mg^2+^ is likely to alleviate the Mg^2+^-mediated inhibition of RyRs. This decreased inhibition could potentially enhance RyR-mediated CICR.

Glycolysis plays a crucial role in insulin secretion. Research has shown that several glycolytic intermediates activate or positively modulate RyR2, with fructose-1,6-diphosphate (FDP) being the most potent [115]. Other active intermediates include glucose-1-phosphate, fructose-6-phosphate, and glucose-6-phosphate. During glucose stimulation, these intermediates likely sensitize RyR2, enhancing CICR.

Glucose stimulation also increases the concentration of arachidonic acid [77] and cADPR [116], which are positive modulators of CICR through RyRs [39]. Glucose metabolism elevates the concentration of long-chain acyl CoA, which can sensitize CICR through RyR2 [117,118].

## 11. Role of RyR-Mediated CICR in Regulating Somatostatin Secretion from δ-Cells

RyR-mediated CICR plays a crucial role in glucose-stimulated somatostatin secretion from mouse δ-cells. Inhibitors of RyRs, such as ryanodine and dantrolene, suppress this secretion [80,119]. In these cells, Ca^2+^ entry through VGCCs triggers CICR via RyRs [80,119]. Mouse δ-cells specifically express the RyR3 receptor, but not RyR1 or RyR2 [80]. A tight functional coupling exists between R-type VGCC and RyR3 to mediate CICR in these cells [80]. Notably, human δ-cells differ from their mouse counterparts, predominantly expressing RyR2 with minimal, if any, RyR3.

Vergari et al. described a RyR3-mediated CICR mechanism in δ-cells driven by Na^+^ [120]. Glucose metabolism and insulin signaling increase Na^+^ entry into δ-cells, likely via sodium–glucose cotransporters or other Na^+^-coupled transporters. Elevated [Na^+^]_i_ activates an intracellular Na+/Ca^2+^ exchanger (functionally analogous to NCLX) and generates a small initial increase in [Ca^2+^]_i_. This Ca^2+^ signal triggers CICR via RyR3, amplifying the Ca^2+^ response and stimulating somatostatin secretion [120].

## 12. Role of RyR-Mediated CICR in Regulating Glucagon Secretion from α-Cells

Glucagon serves as a critical counterregulatory hormone during hypoglycemia. α-cells secrete glucagon maximally when glucose concentrations fall below physiological levels (e.g., 1–3 mM), with low glucose (<4 mM) acting as the primary stimulus. These cells exhibit distinct electrophysiological properties, including low K-ATP channel conductance and Ca^2+^-dependent electrical activity mediated by L-type VGCCs. Notably, while L-type VGCC inhibitors suppress electrical activity, they do not impair glucagon secretion. In contrast, pharmacological blockade of P/Q-type VGCCs with ω-Agatoxin IVA robustly inhibits low-glucose-induced glucagon release [121].

Further experiments by Acreman et al. demonstrated that glucagon secretion under low-glucose conditions is sensitive to ER Ca^2+^ store depletion (cyclopiazonic acid) and RyR inhibition (10 μM ryanodine) [121]. Their findings support a model wherein P/Q-type VGCCs, but not L-type VGCCs, are functionally coupled to RyRs. Specifically, Ca^2+^ influx through P/Q-type channels triggers CICR from RyRs, amplifying Ca^2+^ signals necessary for glucagon exocytosis. This mechanistic uncoupling between L-type VGCC-driven electrical activity and RyR-mediated CICR highlights a specialized signaling pathway in α-cells, ensuring precise glucagon release during hypoglycemia.

## 13. Role of RyRs in Mediating Store-Operated Ca^2+^ Entry (SOCE)

Activation of the RyRs of β-cells triggers Ca^2+^ entry through some TRP-like channels in the plasma membrane [39,42]. Multiple mechanisms are involved in mediating such Ca^2+^ entry. One of them is SOCE, which involves the filling state of the ER Ca^2+^ store, STIM1, Orai1, and some of the TRP channels [122]. β-cells express several TRP channels, some of which are molecular components of SOCE channels [123]. These cells express both RyR1 and RyR2 [54,71]. RyR2 plays an important role in mediating SOCE since deletion of the Ryr2 reduces SOCE [124,125]. In these cells, RyR2 is usually more abundant than RyR1, but some conditions that induce ER stress increase the expression of RyR1 [54]. This leads to leakage of Ca^2+^ from the ER through RyR1, depletion of the ER Ca^2+^ store, SOCE and [Ca^2+^]_i_ oscillation by subthreshold glucose concentrations [54].

RyR activation also triggers Ca^2+^ entry by a mechanism that is independent of the filing state or the ER [42]. Even when the ER Ca^2+^ store is emptied, activation of the RyRs triggers Ca^2+^ entry [42]. RyR activation is required for triggering the Ca^2+^ entry [124]. It appears that conformational changes in the RyRs facilitate the Ca^2+^ entry through direct interactions with the SOCE machinery [126]. In this context, it is noteworthy that RyR1 can interact with TRPC3 and trigger Ca^2+^ entry [127].

Our study shows that activation of RyR2 by several endogenous agonists generated from glucose metabolism activates Ca^2+^ entry through the TRP-like channels in the plasma membrane and depolarizes the plasma membrane potential to the threshold for the activation of VGCCs [42]. It appears that Ca^2+^ release through RyR2 activates TRPM5 in the plasma membrane and Na^2+^ entry through TRPM5 depolarizes the plasma membrane potential [64,128].

## 14. Role of RyR-Mediated CICR in Regulating Electrical Activity of β-Cells

β-cells stimulated by high concentrations of glucose usually show repetitive depolarizations (slow waves). Superimposed on the plateau of the slow waves are bursts of rapid spikes. After these slow waves, there is a silent repolarization or interval phase. The durations of the slow waves and the interval phases may remain regular (“simple bursting”) or may vary (“complex bursting”). In “complex bursting”, the oscillations in the electrical activities show variable patterns, and several patterns of “complex bursting” have been described [129].

The mechanisms underlying “complex bursting” are not clear, but in a theoretical mathematical modeling study, Zhan et al. introduced RyR2 channels into previously known dynamic models of electrical activity in β-cells [130]. According to their model, the level of activation of RyR2 can regulate the bursting periods. The model predicts that moderate activation of RyR2 can change “simple bursting” to a type of “complex bursting” [130]. Periodic activation of RyR2 and the consequent CICR-mediated amplification of Ca^2+^ signals contribute to burst termination by activating the Ca^2+^-activated K^+^ channels, probably the large-conductance Ca^2+^-activated K^+^ channels (BK channels). “Complex bursting” is more effective in increasing average [Ca^2+^]_i_ and insulin secretion [130].

## 15. The Concept of “Leaky RyRs”

“Leaky RyRs” refer to a dysfunction of RyR channels that release Ca^2+^ from the ER inappropriately or in an uncontrolled manner. Leaky RyRs allow Ca^2+^ to escape from the ER into the cytosol. This Ca^2+^ must then be actively pumped back into the ER by SERCA, an ATP-dependent pump. This cycle of leakage and reuptake of Ca^2+^ constitutes a futile Ca^2+^ cycle as it consumes ATP without performing any useful work. Increased ATP consumption can impair cellular energy balance and contribute to pathological outcomes. Leaky RyRs have been linked to clinical disorders like heart failure, muscle weakness, and neurodegenerative diseases [131].

Normally, the binding of FKBP12.6 to RyR2 inhibits RyR2 activity and thereby reduces Ca^2+^ leak from the ER [132]. Oxidation and S-nitrosylation of RyR2 reduce the binding of FKBP12.6 to RyR2, leading to increased ER Ca^2+^ leak through the channel. Islets obtained from human diabetes subjects show increased oxidation, increased nitrosylation of RyR2, and decreased binding of FKBP12.6 to RyR2 [96].

Some gain-of-function mutations in the *RYR2* gene lead to increased Ca^2+^ leak through different mechanisms. The *RYR2*-R2474S and *RYR2*-N2386I mutations cause leaky RyR2 channels by reducing the binding of FKBP12.6 to RyR2. In knock-in mice that express *RYR2*-R2474S or *RYR2*-N2386I, the ER Ca^2+^ store of the islets is depleted due to Ca^2+^ leak through RyR2 channels [96]. Glucose-induced insulin release from islets isolated from these mice is reduced, and the mice exhibit impaired glucose tolerance.

Some people who have a genetic predisposition to catecholaminergic polymorphic ventricular tachycardia (CPVT) have mutations in the *RYR2* gene that lead to leaky RyR2 channels. These patients have impaired glucose tolerance and impaired glucose-induced insulin secretion [96].

## 16. ER Stress and RyRs

ER stress arises from conditions like increased insulin production demands or glucolipotoxicity, leading to accumulation of misfolded/unfolded proteins. This triggers the unfolded protein response (UPR), an adaptive mechanism that becomes pro-apoptotic under sustained stress. Notably, Ca^2+^ dysregulation often mediates ER stress, which contributes to β-cell apoptosis in diabetes pathogenesis [133,134].

ER stress reduces luminal Ca^2+^ stores via RyR-mediated leakage [54,78,135,136]. Both RyR2 activation by misfolded proteins and RyR1 upregulation exacerbate Ca^2+^ efflux [54,78]. This depletion impairs Ca^2+^-dependent chaperones, creating a feedforward loop that amplifies UPR and apoptosis. Pharmacological RyR inhibition protects against ER-stress-induced β-cell death, underscoring its pathogenic role [54,78,136].

Paradoxically, while SERCA inhibition exacerbates ER stress via RyR-driven Ca^2+^ release [136], RyR2 suppression also induces apoptosis through calpain 10 activation [137]. Glucolipotoxicity highlights this bidirectional lethality of RyR modulation, where channel hyperactivity depletes ER Ca^2+^ stores, while its suppression activates an alternative apoptotic pathway—creating a narrow therapeutic window for RyR-targeted interventions [54].

Lessons learnt from the study of disease models:

1. Akita Mice: Mutant proinsulin-induced ER stress causes β-cell apoptosis and diabetes [138]. In these mice, RyR inhibition with ryanodine prevents β-cell apoptosis [78].
2. Wolfram syndrome (*WSF1* mutation): ER Ca^2+^ depletion leads to β-cell death, which is inhibited by RyR inhibitors [135]. Mutations in *WSF1* are associated with a form of young-onset non-autoimmune diabetes [139].
3. *THADA* mutations: A thyroid adenoma-associated (THADA) protein variant binds RyR2, inducing Ca^2+^ leakage that impairs insulin secretion and triggers ER stress-mediated apoptosis [140].

## 17. Role of RyRs in the Pathogenesis of T2DM

**Figure 2:**
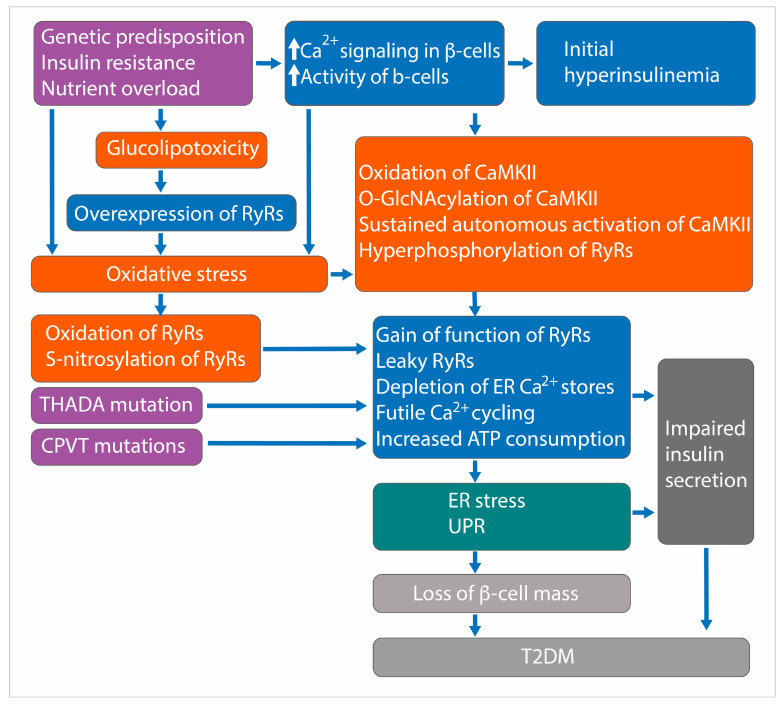
Schematic of the processes involved in the pathogenesis of T2DM. This figure illustrates the sequence of events leading to impaired insulin secretion and loss of β-cell mass in T2DM. Oxidation, S-nitrosylation, and CaMKII-dependent hyperphosphorylation of RyRs result in leaky RyRs, causing futile Ca^2+^ cycling, increased ATP consumption, depletion of ER Ca^2+^ stores, ER stress, and activation of UPR. These processes collectively contribute to impaired insulin secretion and loss of β-cell mass.

The failure of β-cells to compensate for insulin resistance through increased insulin secretion leads to T2DM. This dysfunction involves multiple interconnected processes, including genetic predispositions, metabolic factors, glucolipotoxicity, disrupted signaling, and cellular stresses. Researchers have implicated the RyRs of β-cells and RyR-mediated CICR in the pathogenesis of β-cell defects in T2DM (Figure 2).

### 17.1. Leaky RyRs and Posttranslational Modifications

RyRs can become leaky due to posttranslational modifications such as phosphorylation, oxidation, and nitrosylation. Oxidation and S-nitrosylation of RyR2 lead to increased Ca^2+^ leak and impaired insulin secretion in a mouse model of T2DM [96]. Inhibition of Ca^2+^ leak by the drug S107 shows positive effects on insulin secretion in islets from diabetic patients and murine models of T2DM [96]. Human T2DM islets exhibit increased oxidation and nitrosylation of RyR2, depletion of FKBP12.6, and leaky RyR2 channel.

### 17.2. CaMKII-Mediated Phosphorylation of RyR2

Before the onset of overt T2DM, β-cells experience prolonged periods of increased activity, leading to frequent prolonged elevations of [Ca^2+^]_i_ [141]. This results in increased formation of ROS, oxidation of CaMKII [142], and increased O-GlcNAcylation of CaMKII [143], leading to sustained autonomous activation of CaMKII [87]. Activated CaMKII phosphorylates various proteins, including RyR2. CaMKII-mediated phosphorylation of RyR2 is increased in islets from human T2DM donors and mouse models of T2DM [87]. This phosphorylation leads to gain-of-function in the channel, resulting in increased Ca^2+^ leak from the ER, which can trigger ER stress, and UPR [87].

Studies using RyR2-S2814D knock-in mice, which mimic constitutive phosphorylation of RyR2, show that chronic gain-of-function in RyR2 leads to basal hyperglycemia, impaired GSIS, and glucose intolerance—hallmarks of pre-diabetes and early T2DM [87].

### 17.3. Thyroid Adenoma Associated (THADA) and RyR2 Interaction

*THADA* has been identified as a T2DM-associated gene through genome-wide association studies (GWAS) [140]. The T allele of rs7578597 is considered the risk allele for T2DM [144]. It is strongly associated with T2DM, particularly through maternal inheritance. THADA protein, an ER resident protein, interacts with RyR2, induces Ca^2+^ leak through RyR2, and reduces ER Ca^2+^ stores, triggering ER stress and apoptosis. THADA knock-out in mice enhances β-cell function and reduces β-cell apoptosis, protecting against high-fat high-sucrose and streptozotocin-induced hyperglycemia. Importantly, treatment with alnustone, an inhibitor of THADA protein’s function, ameliorates hyperglycemia in obese mice, suggesting that THADA protein could be a potential target for developing T2DM therapies [140].

### 17.4. RYR2 Mutations and Glucose Intolerance

CPVT patients with *RYR2* mutations, including *RYR2*-R2474S and *RYR2*-N2386I, have been found to have glucose intolerance [96]. Additionally, a missense variant (p.N2291D) in the *RYR2* gene has been identified in individuals with familial T2DM without overt CPVT. The p.N2291D overlaps the RIH (RyR and IP3R homology) domain, indicating that it is crucial for channel function. The p.N2291D variant is in the second mutational hotspot (residues 2246-2534) of the *RYR2* protein. Certain missense mutations in the *RYR2* gene in this hotspot are associated with complete penetrance for glucose intolerance [145].

### 17.5. Other Evidence

Deletion of Ryr2 leads to reduced insulin transcript, content, and glucose-induced secretion [103,124]. RyR2 regulates basal cytoplasmic Ca^2+^ levels and various aspects of Ca^2+^ signaling, such as SOCE and phospholipase C activity [124]. It also plays a role in regulating IRBIT (IP3R-binding protein released with inositol 1,4,5-trisphosphate) levels and activity, which together control insulin production and secretion. Some experiments using an insulin-secreting cell line have shown that glucolipotoxicity increases the expression of RyRs, making cells vulnerable to ER stress [54]. However, it remains unclear whether *RYR* gene expression is increased in human β-cells at any stage of T2DM.

## 18. RYRs and GWAS for T2DM

While GWAS have identified numerous loci associated with T2DM and related glycemic traits, they have not consistently identified variants in the *RYR* genes associated with the disease or its related glycemic traits in large-scale studies [146,147]. For a gene to be identified in GWAS for T2DM, it must have functional polymorphisms that are both common enough in the population and have a sufficient effect size on the disease. *RYR* gene variants associated with T2DM or related glycemic traits are likely rare.

Traditional GWAS focus on common variants (minor allele frequency ≥ 1%), which may not capture rare, functionally significant variants of *RYR* genes. The effect size of common *RYR* variants on T2DM may be small, making them difficult to detect in GWAS with limited sample sizes. Moreover, T2DM is highly polygenic, with many loci contributing small effects, making it challenging to ascertain the contribution of any single gene.

Since GWAS are inadequate for detecting rare variants of *RYRs* associated with T2DM or related glycemic traits, investigators have used other advanced genetic methods. Whole-exome sequencing has identified an atypical missense variant in the *RYR2* gene that co-segregated with T2DM in a family study, associated with glucose intolerance [145].

Family-based association tests using generalized estimating equations (FBAT-GEE) have identified several polymorphisms within the *RYR3* gene associated with the risk of T2DM and age at onset of T2DM [148]. This study found three single-nucleotide polymorphisms (SNPs) significantly associated with T2DM risk, and two other SNPs significantly associated with age at the onset of T2DM.

## 19. Conclusions

Despite their relatively low expression levels in islet cells, RyRs play important roles in Ca^2+^ signaling and hormone secretion. In β-cells, RyRs modulate multiple aspects of Ca^2+^ signaling and Ca^2+^-dependent processes, including the amplification of Ca^2+^ signals via CICR, SOCE, electrical activity, ER stress, UPR, and apoptosis. GLP-1, widely used in diabetes treatment, enhances insulin secretion by promoting RyR-mediated CICR in beta cells. Dysregulation of RyRs, whether through increased phosphorylation or the presence of “leaky” channels, impairs insulin secretion, and certain RyR mutations are linked to impaired glucose tolerance and diabetes.

This review discussed the experimental methods and pharmacological tools used to study RyRs, their complex regulation, and potential reasons for some of the controversies in the field. Further research into the precise roles and regulation of RyRs in islet cells will be essential for understanding their involvement in diabetes pathogenesis and for the development of novel therapeutic strategies.

## Data Availability

Not applicable.

## Abbreviations

The following abbreviations are used in this manuscript:

4-CEP	4-Chloro-3-ethylphenol
4-CmC	4-Chloro-m-cresol
CaMKII	Ca^2+^-calmodulin-dependent protein kinase II
CICR	Ca^2+^-induced Ca^2+^ release
CPVT	Catecholaminergic polymorphic ventricular tachycardia
cADPR	Cyclic ADP-ribose
ER	Endoplasmic reticulum
FKBP12.6	FK506 binding protein 12.6
IP3	Inositol 1,4,5-trisphosphate
IP3R	Inositol 1,4,5-trisphosphate receptor
IP3R3	Inositol 1,4,5-trisphosphate receptor, type 3
MBED9-Methyl-7-bromoeudistomin D	
NO	Nitric oxide
PDE	Phosphodiesterase
PKA	Protein kinase A
RyR	Ryanodine receptor
RyR1	Type 1 ryanodine receptor
RyR2	Type 2 ryanodine receptor
RyR3	Type 3 ryanodine receptor
T2DM	Type 2 diabetes mellitus
*THADA*	Thyroid adenoma associated
UPR	Unfolded protein response
VGCC	Voltage-gated Ca^2+^ channel
